# Epidemiology of Tick-Borne Relapsing Fever in Endemic Area, Spain

**DOI:** 10.3201/eid2605.190745

**Published:** 2020-05

**Authors:** María Carmen Domínguez, Salvador Vergara, María Carmen Gómez, María Esther Roldán

**Affiliations:** Hospital de la Merced, Osuna, Spain (M.C. Domínguez, M.C. Gómez, M.E. Roldán);; Hospital del Tomillar, Seville, Spain (S. Vergara)

**Keywords:** Borrelia hispanica, tick-borne relapsing fever, endemic borreliosis, Jarisch-Herxheimer reaction, bacteria, arthropodborne disease, zoonoses, Spain, vector-borne infections

## Abstract

Tick-borne relapsing fever (TBRF) is caused by spirochetes of *Borrelia* bacteria. We collected data on all TBRF cases in a TBRF-endemic area in southwest Spain during 1994–2016. We analyzed data from 98 patients in whom TBRF was diagnosed by light microscopy and analyzed the relationship between climatic data and TBRF incidence. Most cases occurred a rural environment during summer and autumn. We describe demographic, epidemiologic, clinical, and analytical characteristics, treatment, and occurrence of Jarisch-Herxheimer reaction. Most patients had fever and headache, and laboratory test results included elevated C-reactive protein, thrombocytopenia, and neutrophilia. No patients died, but 10.1% had Jarisch-Herxheimer reaction. *B. hispanica* was the infecting species in 12 cases with PCR results. Clinicians often do not suspect TBRF because clinical signs and symptoms vary; therefore, it is likely underdiagnosed, even in disease-endemic areas.

## Abstract 2

Incidence of this disease increased over time; peak incidences were observed in 2011, 2014, and 2015.

Tick-borne relapsing fever (TBRF) is a zoonosis caused by spirochetes of the genus *Borrelia*. The arthropod vector is a soft tick of the genus *Ornithodoros*, which is endemic to America, Africa, Asia, the Near East and Middle East, and the Iberian Peninsula in Europe (*1*). In each region, a specific relationship exists between *Ornithodoros* spp. and *Borrelia* spp. To date, 23 TBRF-related *Borrelia* species have been confirmed, but additional species are proposed (*2*). *B. hispanica* is the primary TBRF-related *Borrelia* species identified in Spain (*3*,*4*), where it is transmitted mainly through the bite of *O. erraticus* ticks (*5*) but also can be transmitted by *O. occidentalis* ticks (*4*). *B. hispanica* also has been found in Portugal (*6*), Morocco (*4*,*7*,*8*), and Tunisia (*4*).

TBRF is a notifiable disease in Spain and is considered endemic, specifically in Andalusia in southern Spain. During 2003–2017, a total of 85 TBRF cases were reported (*9*), which represented 11% of all reported tickborne diseases, including Lyme disease and Mediterranean exanthematic fever. The areas in Andalusia most affected by TBRF were Seville (67%), Malaga (17.6%), and Cordoba (10.6%). However, the incidence of TBRF could be underestimated because it is difficult to diagnose and because patients and healthcare professionals suspect it less frequently than other tickborne diseases (*9*).

Serologic tests are not useful in diagnosing TBRF because of the antigenic variation of the bacterial variable membrane proteins and cross-reactions with other diseases, such as syphilis and Lyme disease. Hence, these tests are not recommended for routine diagnosis (*10*). Other diagnostic methods, such as molecular detection or isolation of *Borrelia* in special media, usually are available only in reference centers. Demonstration of *Borrelia* in peripheral blood is the definitive method of diagnosis but requires a high degree of experience (*11*).

The pathogenesis of TBRF ranges from a mild to a severe disease, depending on the host and the infecting *Borrelia* species (*12*), and the disease has a broad spectrum of clinical signs and symptoms. Within a few hours of receiving the first dose of antimicrobial drugs to treat TBRF, patients can experience Jarisch-Herxheimer reaction (JHR), an acute exacerbation of symptoms (*13*).

We studied cases of TBRF diagnosed during 1994–2016 at La Merced Hospital, located in southern Spain. Our aim was to enhance knowledge of TBRF epidemiology, clinical and treatment aspects, and risk factors for JHR. In addition, we studied the relationship of this disease and its associated environmental factors.

## Methods

### Patients, Study Design, and Setting

**Figure 1:**
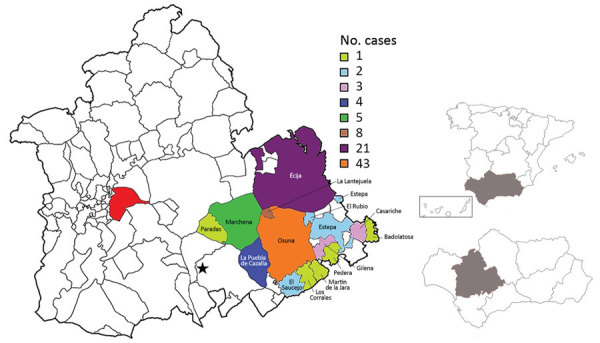
Geographic distribution of tick-borne relapsing fever cases, Spain, 1994–2016. Red indicates city of Seville; star indicates Morón de la Frontera meteorological station. Inset maps show locations of southwestern Spain and Seville Province (gray shading).

We conducted an observational cross-sectional study of all confirmed cases of TBRF diagnosed at La Merced Hospital, a 240-bed public hospital, in Osuna, Spain. We included all patients diagnosed with >1 episode of TBRF during December 1994–December 2016. The area of southern Spain served by this healthcare facility (Figure 1) has a population of ≈174,000.

### Diagnosis of TBRF

Diagnosis of TBRF was made by visualization of *Borrelia* spp. in thick or thin blood smears stained with 10% Giemsa and then examined by optical microscopy. We extracted blood during febrile attacks. Spirochetemia was quantified by light microscopy, at 1,000× magnification, as follows: >5 spirochetes in each field of vision was 3+; 1–5 spirochetes in each field of vision was 2+; <1 spirochete in each field of vision was 1+. During 2013–2014, we sent *Borrelia–*positive samples to the National Microbiology Center (Madrid, Spain) for species identification by multiplex PCR.

### Variables and Definitions

The principal variable was confirmation of TBRF by direct diagnosis. Epidemiologic variables collected included patient age, sex, town of origin, occupation, hobbies, contact with rodents. Other variables recorded were those associated with underlying conditions, such as diabetes mellitus, arterial hypertension, chronic obstructive pulmonary disease, chronic liver disease, chronic kidney disease, cancer, neurologic disease, chronic digestive pathology, cutaneous ulcer, chronic heart failure, HIV infection, transplant recipient, and pregnancy. In addition, variables related to TBRF were recorded, including date of diagnosis, symptoms, remembered antecedent of tick bite, duration of clinical symptoms before diagnosis, main clinical signs, hospitalization, treatment, JHR, and outcome. We analyzed determinants gathered in blood samples, including hemogram and coagulation; serum, including basic biochemistry, C-reactive protein, and liver, renal, and cardiac profiles; and cerebrospinal fluid (CSF), including leukocyte count, percentage of lymphocytes, glucose, and proteins. We used the first determinant collected after symptom onset.

### Meteorologic Conditions

We obtained monthly meteorologic statistics from the records of La Agencia Estatal de Meteorología (http://www.aemet.es/en/portada) of Spain during 1994–2016. We included mean temperature (°C) and percent humidity collected on day 7, 13, and 18 each month at the weather station at Morón de la Frontera (37°9′49.8767ʺN, 5°36′40.5219ʺW). The station is 87 m above sea level and 40 km from Osuna (Figure 1). 

### Statistical Methods

We described cases of TBRF by using epidemiologic and clinical variables and investigated the relationship between environmental factors and TBRF incidence by using a mixed model. We performed calculations by using R software (https://www.r-project.org). We also investigated the risk factors of JHR by performing univariate comparisons of patients who experienced JHR and those who did not by using χ^2^ or Fisher exact test, as needed, for categorical variables and Student *t*-test or Mann-Whitney *U*-test for continuous variables, as appropriate. To control for confounding factors, we performed a multivariate logistic regression analysis by using JHR as the dependent variable. We performed statistical analysis by using SPSS 21.0 (IBM, https://www.ibm.com). We express continuous variables as median and interquartile range (IQR) and categorical variables as number and percent.

### Ethics Approval

The study was designed and performed according to the World Health Association’s Declaration of Helsinki (https://www.wma.net) and was approved by the ethics committee of La Merced Hospital. Because it was a retrospective study, consecutive blinded case numbers were assigned for medical histories taken from patient records to ensure a safe dissociation and prevent identification of patients included.

## Results

### Incidence of TBRF

**Figure 2:**
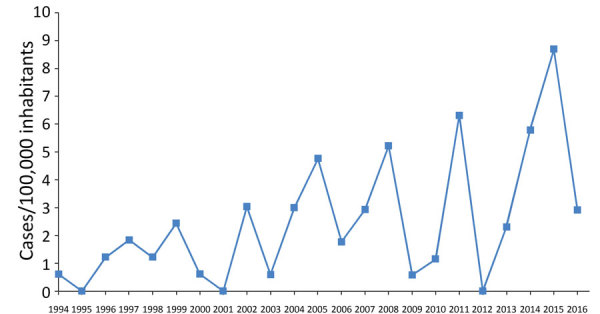
Incidence of tick-borne relapsing fever in Spain, 1994–2016.

We calculated the annual incidence of TBRF cases by analyzing data from 98 case-patients included in our final cohort (Figure 2). TBRF incidence increased over the study period and showed considerable variability between years. The highest incidence rates of TBRF occurred in 2015 (8.69 cases/100,000 persons), 2011 (6.31 cases/100,000 persons), and 2014 (5.77 cases/100,000 persons). Most cases of TBRF were diagnosed during June–November.

### Diagnosis of TBRF

All TBRF cases in our series were diagnosed by direct visualization of *Borrelia* in blood samples. Spirochetemia quantification was either 1+ or 2+. Blood samples were negative 1–2 days after initial *Borrelia* visualization. Samples collected from 12 patients during 2013–2014 were sent to the National Microbiology Center, where the bacteria species was identified as *B. hispanica* by multiplex PCR targeting the 16S rRNA gene.

### Clinical, Analytical, and Therapeutic Features of TBRF Cases

Among 98 patients with TBRF in our cohort, 55 (56.1%) were men and 43 (43.9%) were women (Table 1), 2 of whom were pregnant. Our data agree with the prevalence of TBRF in young persons; 19% of patients in our cohort were <14 years of age (data not shown). Of note, 80% of case-patients lived in a rural environment, and only 17 (17.3%) reported previous known contact with ticks. In most cases, TBRF diagnosis was made in the emergency department. Median time between the onset of symptoms and diagnosis was 3 days (IQR 2–5 days).

**Table 1:** Characteristics of 98 patients with tick-borne relapsing fever, Spain*

Variable	Value	
Median age, y (IQR)	29 (17–46)
Sex	
M	55 (56.1)	
F	43 (43.9)
Town of residence	
Osuna	40 (40.8)	
Ecija	20 (20.4)	
Other	38 (38.8)
Risk activities	
Farming	15 (15.3)	
Hiking	10 (10.2)	
Hunting	3 (3.1)
Contact with rodents	13 (13.3)
Tick bite	17 (17.3)	
JHR, n = 79	8 (10.1)	
Median time before diagnosis, d (IQR)	3 (2–5)	
Hospitalized	56 (57.1)	
Median length of hospital admission, d (IQR)	5 (4–6)
Antimicrobial drug treatment	
Tetracycline	79 (80.6)	
Macrolides	18 (18.4)	
Cephalosporin	9 (9.2)	
Penicillin G	2 (2)
Duration of antimicrobial therapy, d (IQR)	10 (7–14)
Death	0	
*Values are no. (%) except as indicated. IQR, interquartile range; JHR, Jarisch-Herxheimer reaction. †Except where noted.		

The most common clinical symptoms were fever in 99% of cases, headache in 59%, vomiting in 34.7%, and arthralgias in 30% (Table 2). A lumbar puncture was performed on 12 patients; 5 had no neck stiffness, deterioration of consciousness, or signs of Kernig and Brudzinski, but because they had fever, vomiting, and intense headaches, clinicians decided to extract CSF. Lymphocytic meningitis with moderate to high pleocytosis of mononuclear predominance, elevated proteins, and normal glucose were noted in CSF from 3 patients (Table 3). Meningitis was eventually ruled out in a pediatric patient (case 5), who had mild pleocytosis in the CSF without biochemical alterations. No *Borrelia* were observed in the CSF samples. 

**Table 2:** Main symptoms, signs, and analytical findings of 98 patients with tick-borne relapsing fever, Spain*

Variable	Value
Symptoms	
Fever >38.5°C	97 (99)
Headache	58 (59)
Vomiting	34 (34.7)
Arthralgia	29 (30)
Abdominal pain	28 (28.6)
Myalgia	28 (28.6)
Chills	23 (23.6)
Diarrhea	6 (6.1)
Signs	
Meningeal signs	7 (7.1)
Splenomegaly	7 (7.1)
Hepatomegaly	6 (6.1)
Exanthema	4 (4.1)
Jaundice	3 (3.1)
Petechiae	2 (2)
Conjunctival injection	2 (2)
Analytical findings	
Median platelet count, × 10^3^/mm^3^ (IQR)	63.5 (45.7–87.5)
Thrombocytopenia, platelets <13 × 10^4^/mm^3^	91 (92.9)
Hemoglobin, g/dL (IQR)	13.1 (11.8–14.2)
Anemia	32 (32.6)
Leukocytes, ×10^3^/mm^3^ (IQR)	7.9 (6.6–9.8)
Leukocytosis, >11,500/mm^3^	10 (10.2)
Neutrophils, median (IQR)	79.9 (74.5–86.4)
Neutrophilia, neutrophils >65%	91 (92.9)
Prothrombin activity, median (IQR)	77 (70–87)
Decreased prothrombin activity, <70%	7 (13.7)
Median INR (IQR)	1.2 (1.1–1.3)
INR elevation, >1.51	7 (13.7)
SGOT, U/L (IQR)	25 (20–35)
SGOT elevation, >37 U/L	15 (21.7)
SGPT, U/L (IQR)	25 (17–41)
SGPT elevation, >40 U/L	10 (23.3)
Total bilirubin, mg/dL (IQR)	1.1 (0.6–1.9)
Elevated bilirubin, >1.2 mg/dL	37 (37.8)
Lactate dehydrogenase, U/L(IQR)	397.5 (320.7–481.7)
Elevated LDH, >460 U/L	16 (30.8)
Creatinine, mg/dL (IQR)	0.94 (0.79–1.15)
Creatinine elevation, >1.2 mg/dL	15 (15.3)
Creatine kinase, U/L (IQR)	41 (27–75)
Elevated creatine kinase, >195 U/L	3 (14)
C-reactive protein, mg/L (IQR)	254.5 (218.2–335.3)
Elevated C-reactive protein, >5 mg/L	98 (100)
*Values are no. (%) except as indicated. INR, international normalized ratio; IQR, interquartile range; SGOT, serum glutamic-oxaloacetic transaminase; SGPT, serum glutamic-pyruvic transaminase.		

**Table 3:** Findings from cerebrospinal fluid collected from 12 patients with tick-borne relapsing fever, Spain

Case no.	Patient age, y	Protein, mg/dL	Glucose, mg/dL	Leukocytes/µL	Lymphocytes, %	Meningitis
1	50	174	48	1,271	57	Y
2	6	130	61	1,760	90	Y
3	21	91	49	521	90	Y
4	17	74	62	0	0	N
5	5	32	47	130	80	N
6	42	23	74	1	100	N
7	14	20	57	0	0	N
8	19	20	62	4	75	N
9	28	20	49	4	75	N
10	26	19	60	0	0	N
11	32	13	64	1	100	N
12	12	12	78	3	100	N

All 98 cases exhibited elevated C-reactive protein, and 92.9% had thrombocytopenia and neutrophilia, most without leukocytosis, likely due to compensatory reduction of lymphocytes. Anemia was detected in 32 cases, 4 of which were moderate (hemoglobin <10 g/dL). Pancytopenia was observed in 5% of cases, 23% had hypertransaminasemia, and 38% had hyperbilirubinemia.

Regarding treatment, 91 (93%) patients received 1 antimicrobial drug and 7 (7%) received >1 antimicrobial drug. Tetracycline was the first choice in adults and macrolides in persons <14 years of age. The duration of treatment was 10 days (IQR 7–14 days). We observed no notable difference between patients who received >10 days of antimicrobial therapy and those receiving <10 days of treatment (data not shown).

Eleven case-patients had >2 episodes of fever; some had as many as 4. Most recurrences of fever were caused by delay in diagnosis and treatment, except for 1 patient, 7 years of age, who received oral erythromycin for 10 days and was readmitted with the same symptoms 2 weeks later. We detected 2 reinfections: 1 in a self-described farmer and hunter who was affected in 1997 and 2005, and 1 in a hiker who was affected in 2011 and 2013.

We noted 5 clusters of TBRF; 4 occurred in family groups, and 1 involved persons with a common social activity. A 2015 cluster involved 5 family members, a 2014 cluster affected 2 family members, and 3 family members were involved in each cluster noted in 2004 and 2011. Another 2014 cluster occurred among 3 persons who all reported hunting in Osuna, Seville. 

### Correlation between Meteorologic Conditions and TBRF Incidence 

We established a mixed model in which the temperature, humidity, and month have been established as fixed effects and the year as a random competent. When applying the different models, we found that humidity had little effect and that the risk for tick bites was dependent on the temperature and month. Bites were more frequent during summer and autumn months, when higher temperatures were recorded.

### Risk Factors for JHR

Of 98 TBRF cases, we analyzed JHR risk factors for 79 patients, 8 (10.1%) of whom had JHR symptoms, including chills, worsening fever, tachycardia, hypotension, and anxiety, a few hours after starting antimicrobial drug treatment. In our analysis, male sex was a risk factor (8.9%) for JHR compared with female sex (1.3%; p = 0.13) and length of admission (median 5 days; IQR 4–7 days) was a statistically significant risk factor for JHR in univariant analysis (Table 4). We saw no relationship between specific antimicrobial drugs and JHR. In multivariant analysis, we found no variables independently associated with JHR (Table 4).

**Table 4:** Factors associated with risk for JHR among 79 cases of tick-borne relapsing fever, Spain*

Variable	Value			p value†	Adjusted OR (95% CI)	p value‡
Sex				0.13		NS
F	1 (1.3)					
M	7 (8.9)					
Median age, y (IQR)				0.86		NS
Y	28.6 (17.3–46.3)					
N	25.5 (18.9–48.7)					
Rural environment				0.57		NS
Y	7 (8.9)					
N	1 (1.3)					
Contact with rodents				0.11		NS
Y	3 (3.8)					
N	5 (6.3)					
Hepatomegaly				0.46		NS
Y	0					
N	8 (10.1)					
Splenomegaly				0.49		NS
Y	1 (1.3)					
N	7 (8.9)					
Jaundice				0.9		NS
Y	0					
N	8 (10.1)					
Petechiae				0.9		NS
Y	0					
N	8 (10.1)					
Meningeal signs				0.9		NS
Y	0					
N	8 (10.1)					
Exanthema				0.28		NS
Y	1 (1.3)					
N	7 (8.9)					
Tick bite				0.17		NS
Y	3 (3.8)					
N	5 (6.3)					
Tetracycline				0.54		NS
Y	7 (8.9)					
N	1 (1.3)					
Cephalosporin				0.31		NS
Y	2 (2.5)					
N	6 (7.6)					
Macrolides				0.35		NS
Y	1 (1.3)					
N	7 (8.9)					
Median length of clinical signs, d (IQR)§				0.36		NS
Y	3 (2–5.8)					
N	3 (1.3–4)					
Median length of hospital admission, d (IQR)				0.004	0.56 (0.29–1.02)	0.057
Y	5 (4–7)					
N	3 (3–3.3)					
Median duration of antimicrobial therapy, d (IQR)				0.55		NS
Y	10 (8–14)					
N	10 (9–11.8)					
*Values are no. (%) patients with JHR except as indicated. IQR, interquartile range; JHR, Jarisch-Herxheimer reaction; NS, not statistically significant; OR, odds ratio. †Univariant analysis calculated by using χ^2^ or Fisher exact test. ‡Multivariant analysis calculated by using Student *t-*test or Mann-Whitney U*-*test. §Values represent findings from 36 patients with known length of clinical signs.						

## Discussion

We analyzed 98 cases of TBRF identified during a 23-year period from a *Borrelia-*endemic area of Spain. The highest incidence rates were in 2015 (8.69/100,000 persons), 2011 (6.31/100,000 persons), and 2014 (5.77/100,000 persons); no cases were reported in 1995, 2001, or 2012 (Figure 2). The highest number of cases originated in Osuna (43 cases), Ecija (21 cases), La Lantejuela (8 cases), Marchena (5 cases), and La Puebla de Cazalla (4 cases); other areas had <3 cases (Figure 1).

The TBRF incidence trended upward during the study period, probably because of increased awareness of the disease. The peaks observed in 2015, 2014, and 2011 can be justified in part by outbreaks of TBRF among persons in family groups or participating in same social activity. We do not know why years of greater incidence are interspersed among others of little (2009, 2010) or no (2012) incidence. We have not sampled *Ornithodoros* ticks to evaluate densities and infection rates, nor have we collected samples from small mammals to investigate the reservoir of *Borrelia* spp. 

We studied climatic conditions during 1994–2016 and identified a direct correlation of TBRF incidence with increased temperatures during the summer and autumn. Higher TBRF incidence rates during the warmest months can be explained by increased activity of the tick vectors and increased exposure of the general population to those vectors, both of which increase the possibility of tick bites in humans (*8*).

We used Giemsa staining of thin and thick blood films to identify *Borrelia*, but a density of >10^4^ spirochetes/1 mL of blood is needed for definitive diagnosis in thick films and >10^5^ spirochetes/1 mL of blood for thin films and microscopic visualization is not a reliable diagnostic method for TBRF (*13*). Larsson et al. (*14*) suggested the sensitivity of the method could be increased by performing a double centrifugation of the blood sample inoculated in a tube with heparin. The application of phase contrast or dark field microscopy directly on a 10× blood dilution also could be useful for cases of spirochetemia (*10*). Where it is technically possible, PCR is replacing microscopic visualization for diagnosis (*7*,*15*,*16*), but Hospital de la Merced is a small hospital in a rural area and does not have in-house PCR resources.

The most prevalent, and probably the only, *Borrelia* species in the environment in this area is *B. hispanica*, which usually causes mild infection. Patients with TBRF in our hospital had mainly nonspecific infectious clinical signs, such as fever and headache, and none died. Meningitis and facial palsy are the most frequent neurologic complications associated with TBRF (*17*). Only 3 (3.06%) patients in our series had lymphocytic meningitis, and we did not observe any instances of facial palsy. However, meningitis and encephalitis have been reported in infections caused by other *Borrelia* species, including *B. crocidurae* (*18*), *B. duttoni*, and *B. turicatae* (*17*).

We did not observe additional clinical complications in pregnant women, but Rustenhoven-Sappan et al. (*19*) reported that pregnant women have higher spirochete loads and more severe symptoms than women who are not pregnant. Some evidence also suggests that spirochetes can cross the placenta and cause neonatal relapsing fever (*20*). However, we only had 2 TBRF cases in pregnant women, so the lack of clinical complications in this group likely can be attributed to the small sample size in our series.

The most common abnormality in blood cell count we noted in laboratory findings was thrombocytopenia, especially during the early stage of infection, with a median of 63,500 platelets/mm^3^ in the hemogram. Thrombocytopenia has been reported in *B. crocidurae* (*12*), *B. hermsii* (*21*), and *B. hispanica* (*22*–*24*) infections, but not with *B. burgdorferi* (*25*). Thrombocytopenia could be explained by the phenomenon of bacteria binding directly to platelets during the infection, which results in increased platelet destruction, prolonged bleeding, or endothelial injury, the degree of which correlates with the degree of spirochetemia (*21*).

We observed anemia in 33% of patients at TBRF diagnosis with hemoglobin values <11.8 g/dL and hematocrits <35%, likely caused by erythrocyte rosetting. The aggregation of red blood cells around *B. hispanica* has been observed in vitro by Guo et al. (*26*) and has been described in infections with *B. duttonii* and *B. coriaceae* (*26*). Erythrocyte rosetting also has been reported by Shamaei-Tousi et al. (*12*) in an animal model infected with the *B. crocidurae*. 

We also detected neutrophilia that did not produce leukocytosis because of compensatory lymphopenia. López et al. (*27*) studied an animal model with Rhesus macaques infected with *B. turicatae* and observed notable changes in the differential leukocyte count. They found a relative increase in the neutrophil percentage for several days after leukopenia and mild increases in monocytes several days after the initial episode of leukopenia. They noted these abnormalities to some degree in all animals studied, and the neutrophilia coincided with spirochetemia (*27*). 

Another finding that deserves attention is pancytopenia, a potentially serious complication, which we observed in 5% of TBRF cases. Fortunately, severe pancytopenia is a rare complication described in only a few cases (*13*,*24*).

All patients in our study demonstrated known TBRF biochemical alterations in the acute phase, including high levels of C-reactive protein (*22*–*24*), mild hypertransaminasemia (*13*,*28*,*29*), and hyperbilirubinemia (*29*,*30*). Less frequently, elevated lactate dehydrogenase and creatinine kinase also have been reported (*22*); we observed elevated lactate dehydrogenase in 31% of cases in our study and elevated creatinine kinase in 15%. 

*Borrelia* spirochetes are susceptible to penicillin and other β-lactam antimicrobial drugs and to tetracyclines and macrolides. Most experts recommend a dose of 100 mg doxycycline twice a day or 500 mg tetracycline four times a day for 7–14 days (*1*). Erythromycin or chloramphenicol are the most used alternatives (*1*). Parenteral therapy with ceftriaxone is recommended for patients with central nervous system involvement or severe multisystem disease (*1*). Fluoroquinolones are not recommended for treating patients with TBRF (*29*). In our experience, we treated patients with some of the recommended regimens over a median of 10 days, and they had adequate, early responses to treatment. A shorter regimen might be equally effective for treating TBRF.

We observed JHR in 10.1% of patients. Our patients did not have severe JHR, but severe cases have been reported (*31*), including elevated cardiac troponin in pregnant women (*23*,*28*) and, more frequently, in children (*19*,*23*). However, neither of the 2 pregnant women in our TBRF cohort developed JHR.

Immunity to *B. hispanica* after infection is not permanent, so patients can be reinfected later. We detected 2 cases of reinfection. Reinfections with *B. crocidurae* also have been observed in patients in Senegal (*32*), demonstrating the importance of informing patients of this aspect of TBRF.

In conclusion, we analyzed epidemiologic data of 98 TBRF cases diagnosed in southern Spain during 1994–2016. In addition to information on the *Borrelia* life cycle, we report clinical signs, laboratory findings, prognosis, complications, and TBRF diagnosis, in patients at a rural hospital. Because clinical signs and symptoms of TBRF cover a wide range and incidence is low, clinicians have a low suspicion index for this disease. The most frequent symptoms are fever and headache and the most frequent analytical alterations are thrombocytopenia and neutrophilia without leukocytosis, in addition to the elevated C-reactive protein during the acute phase. TBRF occurs in rural environments, mainly in summer and autumn. We saw no evidence of a climate-associated increase in infection risk over the 23-year period. TBRF usually is not a severe disease in patients in this region of Spain, and they respond well to treatment. Because the spirochetemia phase is short and laboratory diagnosis is exclusively dependent on the observer, we believe TBRF is underdiagnosed, even in areas where suspicion should be relatively high. Addition of routine molecular techniques to detect spirochetes could eliminate these diagnostic doubts in rural areas.
